# Transgenic soybean overexpressing *GmSAMT1* exhibits resistance to multiple‐HG types of soybean cyst nematode *Heterodera glycines*

**DOI:** 10.1111/pbi.12566

**Published:** 2016-05-23

**Authors:** Jingyu Lin, Mitra Mazarei, Nan Zhao, Catherine N. Hatcher, Wegi A. Wuddineh, Mary Rudis, Timothy J. Tschaplinski, Vincent R. Pantalone, Prakash R. Arelli, Tarek Hewezi, Feng Chen, Charles Neal Stewart

**Affiliations:** ^1^ Department of Plant SciencesUniversity of TennesseeKnoxvilleTNUSA; ^2^ Biosciences DivisionOak Ridge National LaboratoryOak RidgeTNUSA; ^3^ Crop Genetics Research UnitUSDA‐ARSJacksonTNUSA

**Keywords:** salicylic acid methyltransferase, transgenic soybean, soybean cyst nematode, gene expression, metabolite, female index, yield

## Abstract

Soybean (*Glycine max* (L.) Merr.) salicylic acid methyl transferase (GmSAMT1) catalyses the conversion of salicylic acid to methyl salicylate. Prior results showed that when *GmSAMT1* was overexpressed in transgenic soybean hairy roots, resistance is conferred against soybean cyst nematode (SCN), *Heterodera glycines* Ichinohe. In this study, we produced transgenic soybean overexpressing *GmSAMT1* and characterized their response to various SCN races. Transgenic plants conferred a significant reduction in the development of SCN HG type 1.2.5.7 (race 2), HG type 0 (race 3) and HG type 2.5.7 (race 5). Among transgenic lines, *GmSAMT1* expression in roots was positively associated with SCN resistance. In some transgenic lines, there was a significant decrease in salicylic acid titer relative to control plants. No significant seed yield differences were observed between transgenics and control soybean plants grown in one greenhouse with 22 °C day/night temperature, whereas transgenic soybean had higher yield than controls grown a warmer greenhouse (27 °C day/23 °C night) temperature. In a 1‐year field experiment in Knoxville, TN, there was no significant difference in seed yield between the transgenic and nontransgenic soybean under conditions with negligible SCN infection. We hypothesize that *GmSAMT1* expression affects salicylic acid biosynthesis, which, in turn, attenuates SCN development, without negative consequences to soybean yield or other morphological traits. Thus, we conclude that *GmSAMT1* overexpression confers broad resistance to multiple SCN races, which would be potentially applicable to commercial production.

## Introduction

In the United States, the soybean cyst nematode (SCN) (*Heterodera glycines* Ichinohe) is the most damaging pest to soybean (*Glycine max* [L.] Merr.) (Koenning and Wrather, 2010). SCN‐resistant cultivars are currently the most effective way to manage SCN. However, among a total of 118 SCN‐resistant accessions that have been identified in the USA, few have any resistance to four or more SCN races (Arelli *et al*., 2000). Soybean breeders have identified quantitative trait loci (QTL), which are distributed over the genome. Most SCN‐resistance QTL can be traced to ‘Peking’ PI 437654 and PI 88788 (Concibido *et al*., 2004), which provide some resistance to SCN races 2, 3 and 5 (Guo *et al*., 2005). Genes cloned from two QTL from PI 88788 and cultivar ‘Forrest’ (Cook *et al*., 2012; Liu *et al*., 2012) have provided new insights into understanding of SCN resistance mechanisms. The most well‐known resistance QTL are located on chromosomes 18 (*rhg1*) and 8 (*Rhg4*), which are considered to be the major sources of resistance in soybean cultivars (Concibido *et al*., 2004). The *rhg1* gene is vital for broad SCN resistance (Concibido *et al*., 2004; Guo *et al*., 2005), and the *Rhg4* gene is critical for resistance to race 3 (Concibido *et al*., 2004; Webb *et al*., 1995). A number of minor resistance genes have also been reported, which vary according to germplasm and nematode race (Winter *et al*., 2006). The SCN resistance conferred by *rhg1* in PI 88788 is mediated by a 31‐kb genomic region, where three genes were found to contribute to SCN resistance when expressed simultaneously (Cook *et al*., 2012). High copy numbers of this genomic region resulted in increased basal expression levels of these three genes and SCN resistance (Cook *et al*., 2012). *Rhg4* encodes a serine hydroxymethyltransferase, which plays a critical role in cellular one‐carbon metabolism (Liu *et al*., 2012). However, the mechanism of interactions between soybean and variable SCN HG types remains elusive.

Plant hormone salicylic acid (SA) acts as a regulatory signal to mediate a diverse range of plant defence responses. The SA pathway is primarily effective in mediating resistance against biotrophic pathogens (Gimenez‐Ibanez and Solano, 2013). SA‐treated white clover root prior to inoculation with the clover cyst nematode (*Heterodera trifolii*) resulted in decreased nematode reproduction in this system (Kempster *et al*., 2001). In another system, SA‐deficient mutants of *Arabidopsis thaliana* exhibited increased susceptibility to beet cyst nematode (*Heterodera schachtii*) (Wubben *et al*., 2008). There was evidence showing that the successful parasitism of the nematode required manipulation of genes involved in regulation and biosynthesis of SA and its associated signalling pathway (Matthews *et al*., 2014). It was reported that overexpression of some genes, *AtNPR1, AtGA2* and *AtPR‐5,* which encode specific components involved in SA regulation, synthesis and signalling, respectively, improved the soybean resistance to SCN (Matthews *et al*., 2014). The cyst nematode effector 10A06 was found to interfere with basal defence responses by repressing SA signalling to mediate susceptibility (Hewezi *et al*., 2010).

We previously identified a soybean salicylic acid methyl transferase gene (*GmSAMT1*) as a candidate SCN defence‐related gene (Mazarei *et al*., 2011) and studied SCN race 2 (HG type 1.2.5.7) development in soybean hairy roots overexpressing *GmSAMT1* (Lin *et al*., 2013). *GmSAMT1* encodes an *S*‐adenosyl‐l‐methionine‐dependent salicylic acid carboxyl methyltransferase, which catalyses the methylation of SA to form methyl salicylate (MeSA) (Lin *et al*., 2013). The role of MeSA in plant defence was indicated as a defence signal, which was systemically transported and converted to SA to activate systemic acquired resistance (SAR) (Park *et al*., 2007). The SAMT genes from other plant species, including *Arabidopsis* (Chen *et al*., 2003), rice (Koo *et al*., 2007; Zhao *et al*., 2010) and tobacco (Park *et al*., 2007) are also involved in plant defence. For instance, SAR in response to tobacco mosaic virus (TMV) was found to be blocked when *NtSAMT1* was silenced in primary infected tobacco leaves (Park *et al*., 2007). The transgenic tomato with overexpression of *SAMT* exhibited delayed disease symptoms following infection with the bacterial pathogen *Xanthomonas campestris* pv. *vesicatoria* (*Xcv*) (Tieman *et al*., 2010). Transgenic *Arabidopsis* overexpressing a rice salicylic acid/benzoic acid carboxyl methyltransferase gene *OsBSMT1* was more susceptible to disease caused by the bacterial pathogen *Pseudomonas syringae* or the fungal pathogen *Golovinomyces orontii* than the wild‐type plants (Koo *et al*., 2007). In our prior study, transgenic hairy root with overexpression of *GmSAMT1* in SCN‐susceptible soybean significantly reduced the development of SCN race 2, indicating that *GmSAMT1* played a role in resistance against SCN.

The objectives of this study were to characterize multiple generations of *GmSAMT1* transgenic soybean plants in the greenhouse and field for resistance to SCN, determine whether there were correlations between *GmSAMT1* expression with SCN resistance, key metabolite profiles and seed yield.

## Results

### Stable transgenic soybean with overexpression of *GmSAMT1*

Ten independent transgenic T1 soybean lines were generated, and nine lines were confirmed to contain a single T‐DNA insertion using herbicide‐resistance segregation analysis (Table S1). Subsequently, homozygous T3 transgenic soybean lines were obtained from self‐fertilization and used for further analysis. PCR analysis showed that *GmSAMT1* and *Bar* (bialaphos resistance gene) genes were stably inherited in these soybean lines (Figure S1). The copy number was further confirmed by quantitative PCR (qPCR). The T2 homozygous lines (L5‐5, L20‐3, L56‐3 and L65‐3) contained one copy number of the transgene, which is consistent with the herbicide‐resistance segregation analysis (Table S1). However, based on the qPCR results, the transgenic soybean lines L16‐2 and L16‐3, which were derived from the same event (L16), contained 2 copies of the transgene (Table S2).

### Gene expression analysis of transgenic soybean roots

**Figure 1:**
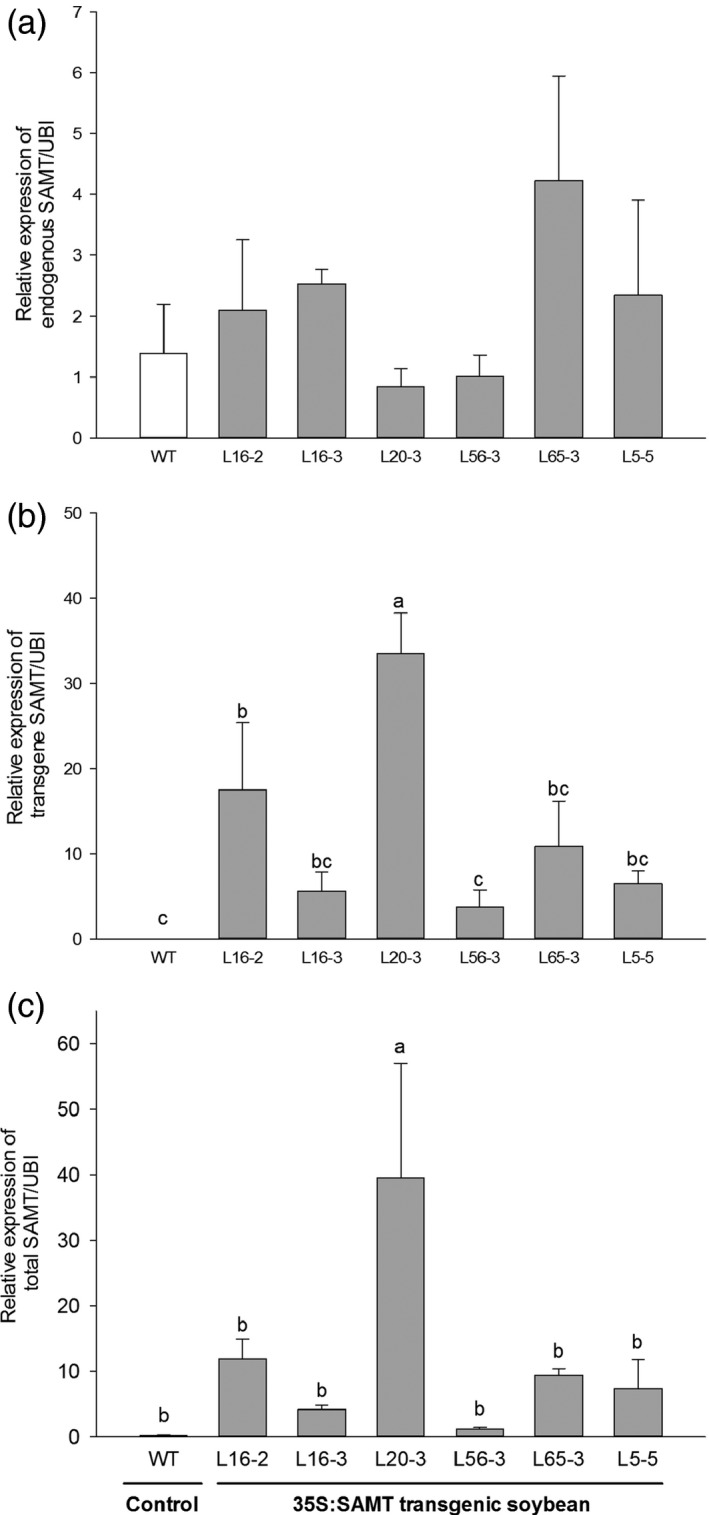
The relative expression of *GmSAMT1* in T3 homozygous transgenic soybean roots determined by qRT‐PCR. (a) The relative expression of endogenous *GmSAMT1*. (b) The relative expression of transgene *GmSAMT1*. (c) The relative expression of total *GmSAMT1*. The relative levels of transcripts were normalized to soybean ubiquitin gene (*GmUBI*). Bars represent mean values of three biological replicates ± standard error. Bars with different letters are significantly different at *P *<* *0.05 as tested by Fisher's least significant difference. Nontransgenic Williams 82 (WT) soybean as control and 35S:SAMT transgenic soybean lines L5‐5, L16‐2, L16‐3, L20‐3, L56‐3 and L65‐3 with overexpression of *GmSAMT1* were studied.

The endogenous relative qRT‐PCR transcript abundance of *GmSAMT1* (Figure S2) among homozygous T3 transgenic soybean lines was invariant among transgenic lines and controls (Figure 1a), whereas the relative expression of the transgene was higher among lines, especially L20‐3 (Figure 1b,c). Line L20‐3 had approximately 226‐fold increased *GmSAMT1* transcript abundance relative to the nontransgenic control. Line L56‐3 had only a 7‐fold increase, whereas several lines (L5‐5, L16‐2, L16‐3 and L65‐3) had moderately high (between 24‐ and 48‐fold) increases compared to nontransgenic control (Figure 1c). There were no steady‐state differences in mRNA abundance of pathogenesis‐related (PR) genes *PR1* (acidic pathogenesis‐related 1) and *PR3* (basic chitinase) among lines (Figure S3).

### Response of transgenic soybean to SCN infection

**Figure 2:**
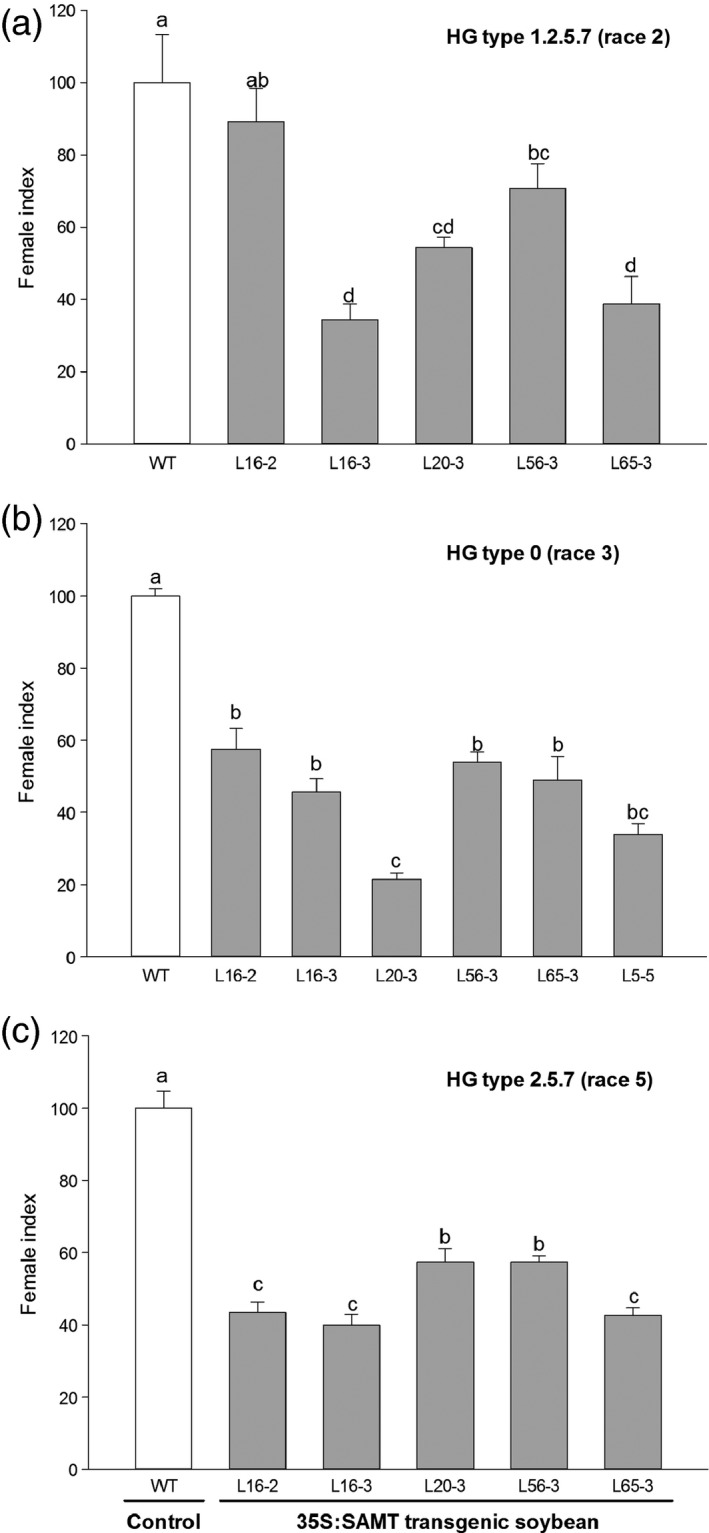
Soybean cyst nematode (SCN) bioassay of T3 homozygous transgenic soybean plants overexpressing *GmSAMT1* gene. SCN HG type 1.2.5.7 (race 2) (a), SCN HG type 0 (race 3) (b) and SCN HG type 2.5.7 (race 5) (c) were used for the SCN bioassay experiments. Bars represent mean values (*n* ≥ 12) of the female index with ± standard error. Bars with different letters are significantly different at *P *<* *0.05 as tested by Fisher's least significant difference. Nontransgenic Williams 82 (WT) soybean as control and 35S:SAMT transgenic soybean lines L5‐5, L16‐2, L16‐3, L20‐3, L56‐3 and L65‐3 with overexpression of *GmSAMT1* were studied.

**Figure 3:**
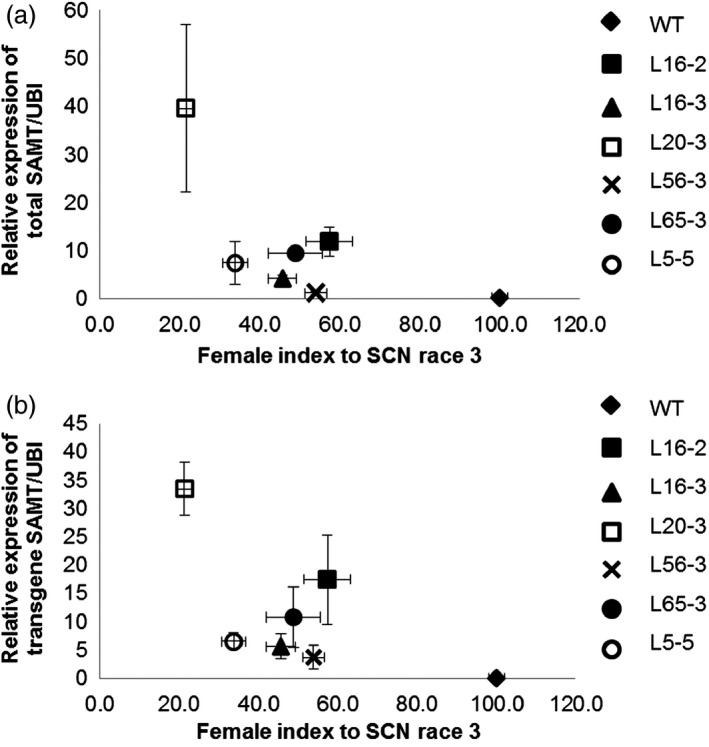
Correlation between relative transcript levels in roots and soybean cyst nematode (SCN) female index. (a) The relative expression of total *GmSAMT1* and female index for SCN HG type 0 (race 3) (*r* = −0.51, *P *=* *0.018). (b) The relative expression of transgene *GmSAMT1* and female index for SCN HG type 0 (race 3) (*r* = −0.56, *P *=* *0.009). The relative levels of transcripts were determined by qRT‐PCR and normalized to soybean ubiquitin gene (*GmUBI*). Nontransgenic Williams 82 (WT) soybean as control and 35S:SAMT T3 homozygous transgenic soybean lines L5‐5, L16‐2, L16‐3, L20‐3, L56‐3 and L65‐3 with overexpression of *GmSAMT1* were studied.

There was no significant difference on the number of eggs per cyst (ranging from 46 to 60 eggs per cyst) between the control and transgenic lines. Therefore, for comparison of the SCN resistance between the control and transgenic lines, the number of cysts from each line was counted for calculation of female index. Six transgenic homozygous T3 transgenic lines with varied transgene expression conferred a range of resistance against three HG types of SCN with decreases in the SCN HG type 1.2.5.7 (race 2) female index, which ranged from 46% to 66% relative to the control (Figure 2a). Transgenic lines also exhibited statistically significant decrease in female index when they were inoculated with HG type 0 (race 3) and HG type 2.5.7 (race 5) SCN. For HG type 0 (race 3), the decrease in the SCN female index ranged from 46% to 79% (Figure 2b). The decrease in the SCN female index ranging from 43% to 60% was also observed for HG type 2.5.7 (race 5) (Figure 2c). There was a significant negative correlation between total *GmSAMT1* expression and SCN HG type 0 (race 3) female index (*r* = −0.51, *P* = 0.018), as well as between transgene expression and SCN HG type 0 (race 3) female index (*r* = −0.56, *P* = 0.009) (Figure 3a,b). No significant correlation was observed between other SCN HG types and the expression of *GmSAMT1*.

### Effect of SCN inoculation on growth of the transgenic soybean plants

**Figure 4:**
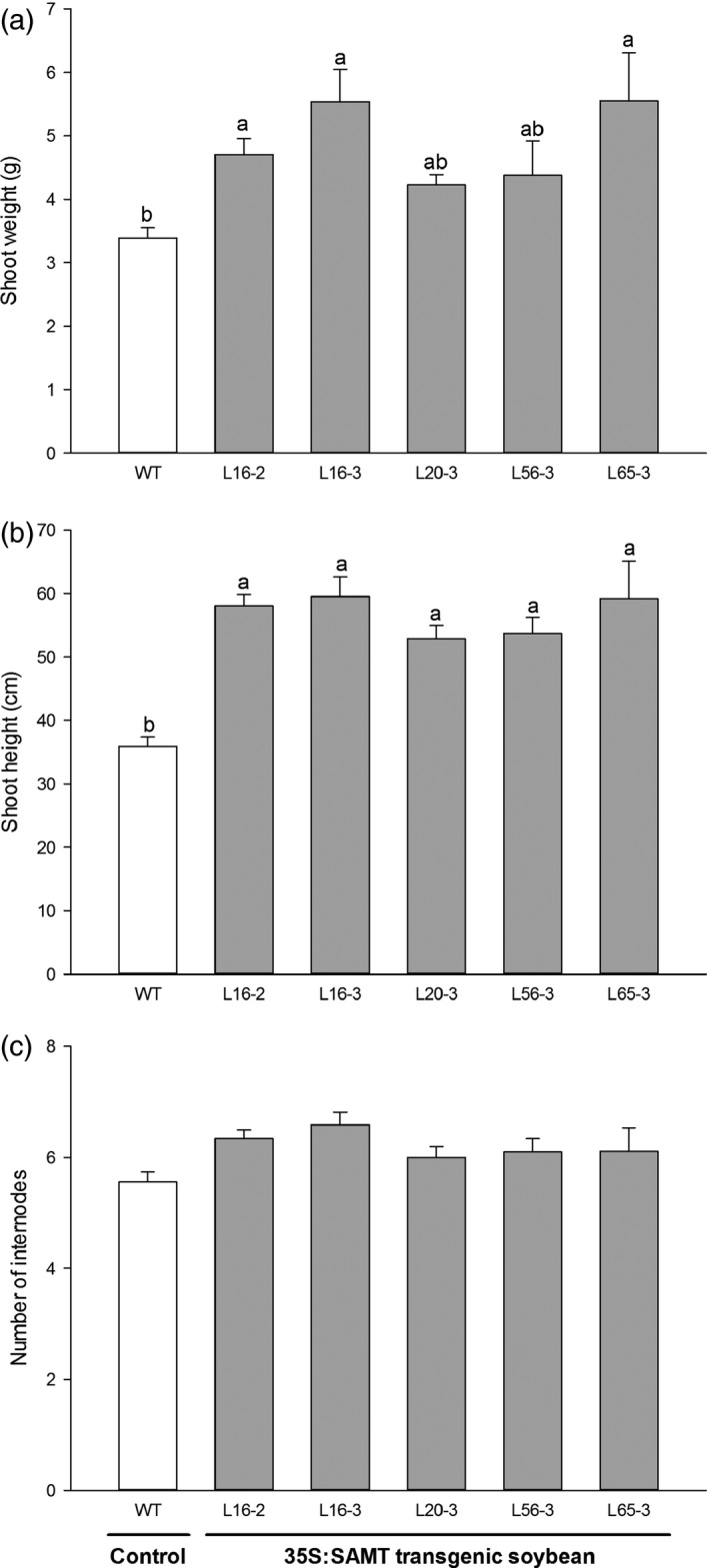
The effect of soybean cyst nematode (SCN) inoculation on the growth of T3 homozygous transgenic soybean plants overexpressing *GmSAMT1* gene. Shoot weight (a), shoot height (b), and number of internodes (c) were examined on the transgenic soybean plants inoculated by SCN HG type 2.5.7 (race 5). Bars represent mean values (*n* ≥ 8) with ± standard error. Bars with different letters are significantly different at *P *<* *0.05 as tested by Fisher's least significant difference. Nontransgenic Williams 82 (WT) soybean as control and 35S:SAMT transgenic soybean lines L16‐2, L16‐3, L20‐3, L56‐3 and L65‐3 with overexpression of *GmSAMT1* were studied.

Three transgenic soybean lines (L16‐2, L16‐3 and L65‐3) had significantly higher fresh shoot weight (ranging from 4.7 g to 5.6 g) compared with control (3.4 g) at 35 days postinoculation with SCN HG type 2.5.7 (race 5) (Figure 4a). All transgenic soybean lines were significantly taller (ranging from 52.9 cm to 59.5 cm) compared with control soybean (35.9 cm) (Figure 4b). There was no significant difference of internode number among lines (Figure 4c).

### Soybean leaf metabolite analysis relative to transgene expression in roots

**Figure 5:**
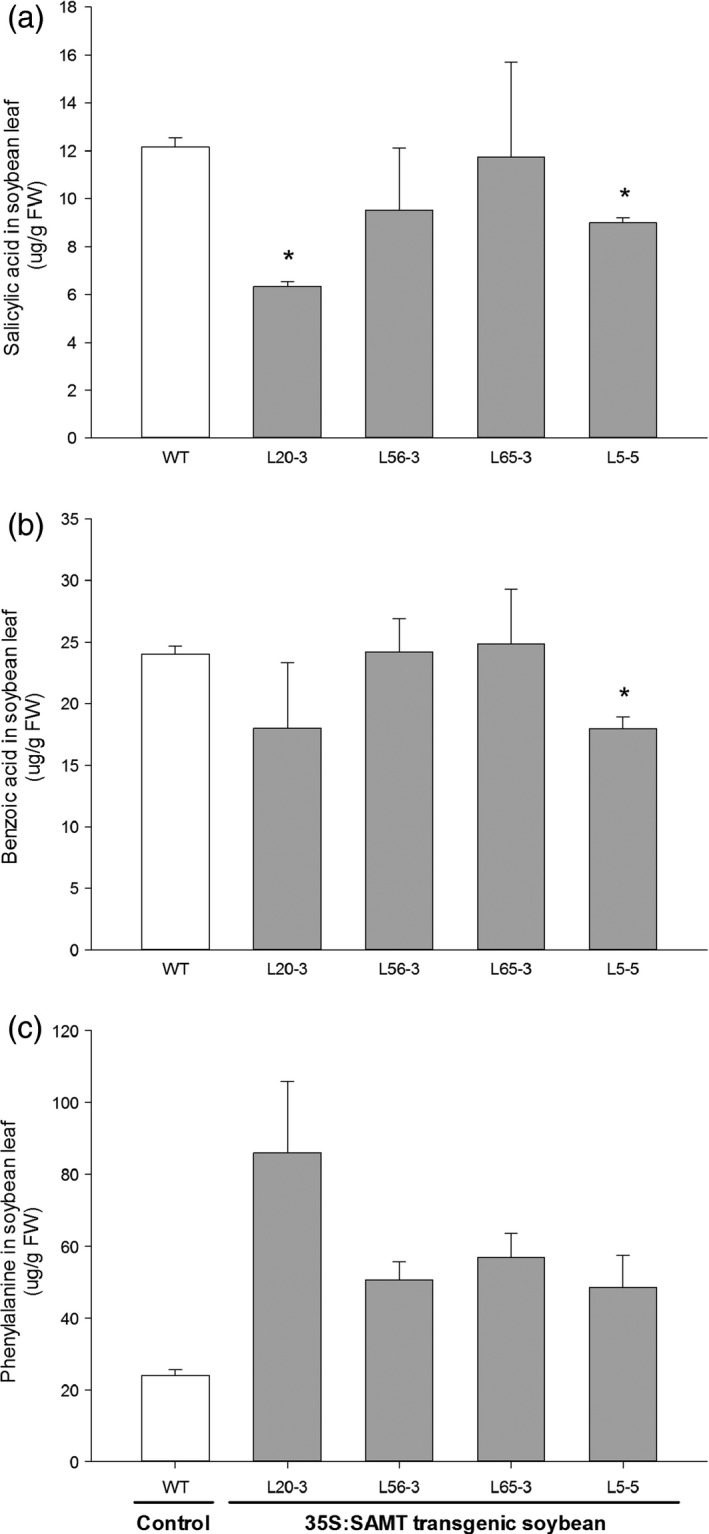
Related metabolites in the T3 homozygous transgenic soybean leaves overexpressing *GmSAMT1*. (a) Salicylic acid, (b) benzoic acid, (c) phenylalanine titers from leaf tissue. Bars represent mean values of two biological replicates with ± standard error. Asterisks indicate significant differences from nontransgenic control plants at *P *<* *0.05 as determined by *t*‐test. Nontransgenic ‘Williams 82’ (WT) soybean as control and 35S:SAMT transgenic soybean lines L5‐5, L20‐3, L56‐3 and L65‐3 with overexpression of *GmSAMT1* were studied.

**Figure 6:**
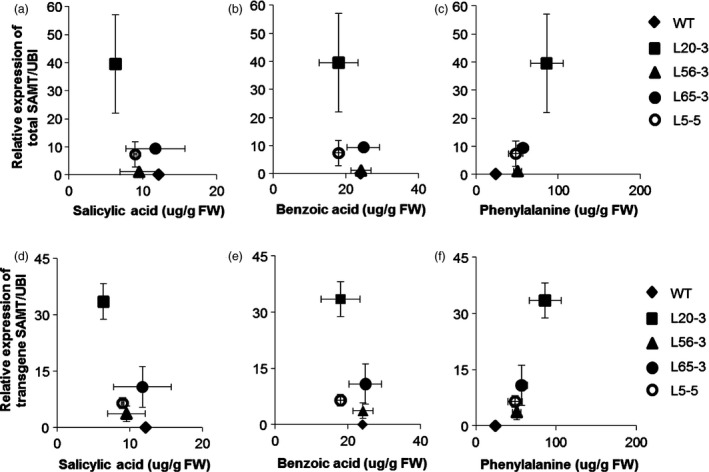
Correlation between leaf metabolites assayed and the relative expression of *GmSAMT1* in roots (a) Correlation between salicylic acid and the relative expression of total *GmSAMT1* (*r* = −0.42, *P *=* *0.116). (b) Correlation between benzoic acid and the relative expression of total *GmSAMT1* (*r* = −0.31, *P *=* *0.258). (c) Correlation between phenylalanine and the relative expression of total *GmSAMT1* (*r* = 0.70, *P *=* *0.004). (d) Correlation between salicylic acid and the relative expression of transgene *GmSAMT1* (*r* = −0.39, *P *=* *0.151). (e) Correlation between benzoic acid and the relative expression of transgene *GmSAMT1* (*r* = −0.37, *P *=* *0.179). (f) Correlation between phenylalanine and the relative expression of transgene *GmSAMT1* (*r* = 0.86, *P *<* *0.001). Horizontal error bars represent the standard error of metabolite amount from two biological replicates. Vertical error bars represent standard error of the relative gene expression level from three biological replicates. Nontransgenic Williams 82 (WT) soybean as control and 35S:SAMT T3 homozygous transgenic soybean lines L5‐5, L20‐3, L56‐3 and L65‐3 with overexpression of *GmSAMT1* were studied.

The highest‐expressing line (L20‐3) had about half SA titer (6.4 μg/g fresh weight) than the nontransgenic control line (12.2 μg/g fresh weight) (Figure 5a). SA was negatively correlated (*r* = −0.42, *P* = 0.116) with *GmSAMT1* transcript abundance (*r* = −0.39, *P *=* *0.151) (Figure 6a,d). The level of benzoic acid (BA), the proposed direct precursor, showed a decreased trend as well (Figures 5b and 6b,e). However, phenylalanine (PA), the key component of the SA biosynthesis pathway, was found to be elevated in the transgenic soybean lines. For example, line L20‐3, which had the highest transgene expression, contained a high concentration of PA (86.0 μg/g fresh weight), compared with the nontransgenic control soybean line (24.0 μg/g fresh weight) (Figure 5c). The expression of total *GmSAMT1* (*r* = 0.70, *P *=* *0.004) and transgenic *GmSAMT1* (*r* = 0.86, *P *<* *0.001) was significantly positively correlated with the amount of PA (Figure 6c,f).

### Greenhouse plant growth and yield

There were no significant seed yield differences in soybean lines and nontransgenic soybean grown in greenhouse conditions under 22 °C day/night temperature (Figure S4). However, under relatively warmer greenhouse conditions (the temperature was about 5 °C higher than the other greenhouse), transgenic soybean lines trended higher seed weight per plant relative to controls (Figure S5a). The seed number per plant for transgenic soybean lines L16‐3 and L65‐3 (109 and 82, respectively) was also higher than nontransgenic soybean (52) (Figure S5c). However, there was no significant difference on the single seed weight between the transgenic soybean lines and nontransgenic soybean in the two greenhouse experiments (Figures S4b and S5b). Among lines, plant morphology appeared invariant.

### Field experiment

Five T4 homozygous transgenic soybean lines overexpressing *GmSAMT1* (L16‐2, L16‐3, L20‐3, L56‐3 and L65‐3) showed no statistically significant differences in plant phenotype (Figure S6), agronomic traits, such as maturity (ranging from 116 days to 124 days), seed yield (ranging from 3840.1  kg/ha to 4282.6 kg/ha), seed size (ranging from 15.16 g to 16.36 g 100‐seed weight), plant height (ranging from 108.4 cm to 114.3 cm) and lodging score (ranging from 1.3 to 2.7) compared with the nontransgenic parent line (‘Williams 82’) and null segregant control lines (Table 1).

**Table 1:** Agronomic trait of transgenic soybeans in the field evaluation

Transgenic soybean lines	Agronomic trait				
	Maturity (day)	Plant height (cm)	Lodging (score)	100‐seed weight (g)	Seed yield (kg/ha)
L16‐2	120.7 ± 2.0	109.2 ± 2.6	1.7 ± 0.3	15.53 ± 0.28	3924.2 ± 170.4
L16‐3	122.0 ± 1.0	109.2 ± 2.2	2.3 ± 0.7	15.16 ± 0.15	3840.1 ± 223.4
L20‐3	121.7 ± 2.3	108.4 ± 2.2	1.7 ± 0.3	16.13 ± 0.45	3896.2 ± 185.1
L56‐3	116.3 ± 2.9	114.3 ± 1.9	1.3 ± 0.3	16.36 ± 0.17	4282.6 ± 210.5
L65‐3	124.0 ± 0	110.1 ± 4.3	2.7 ± 0.9	16.04 ± 0.27	4221.9 ± 462.7
Null Segregant	123.3 ± 1.2	108.0 ± 1.9	3.7 ± 1.3	15.29 ± 0.39	3912.3 ± 238.4
‘Williams 82’	123.0 ± 1.2	109.6 ± 1.5	3.3 ± 0.3	16.07 ± 0.46	3647.3 ± 135.9

The field experiment was set in a randomized complete block design with three replications. Each replicate contained 168 plants. Values are means of three replicates ± standard errors. Lodging score: 1 = excellent (plants erect), 5 = poor (plants lodged); seed yield calculation was based on dry matter seed weight. Statistical significance was tested with one‐way ANOVA, and no significant difference was detected between transgenic and nontransgenic soybean.

## Discussion

The significant diversity of field populations of *H. glycines* combined with limited availability of resistant cultivars could increase the possibility of population shifts over time, resulting in SCN overcoming the limited available resistance in commercial soybean germplasm (Zheng *et al*., 2006). Novel sources of SCN resistance should expand the genetic basis of SCN management, with the emphasis on the molecular mechanisms of infection and resistance. Novel genetic mechanisms for resistance can be deployed via biotechnology and/or breeding, which are critical to affect durable yields in the face of multiple SCN races/HG types.

In our previous study, biochemical assays characterized GmSAMT1 to be a SA methyltransferase (Lin *et al*., 2013). Overexpression of *GmSAMT1* in soybean hairy roots in two susceptible backgrounds exhibited increased resistance up to 46.6% against SCN HG type 1.2.5.7 (race 2) (Lin *et al*., 2013). In the current study, stable *GmSAMT1* of transgenic soybean was produced, and homozygous T3 soybean lines with a range of transgene expression were challenged with multiple‐HG types of SCN. The transgenic soybean line (L20‐3), the line with the highest transgene expression also had the highest SCN resistance against HG type 0 (race 3) (Figure 2b). Lower expressing lines, such as L16‐3 and L65‐3, had relatively higher SCN resistance against HG type 1.2.5.7 (race 2) and HG type 2.5.7 (race 5) (Figure 2a,c). These results suggest *GmSAMT1* expression might play a variable role in resistance against different SCN HG types, which was an unexpected finding of this research. The role played by *SAMT* plant genes in disease resistance against bacterial and fungal pathogens including *Pseudomonas syringae, Golovinomyces orontii* and *Hyaloperonospora arabidopsis* has been previously reported (Koo *et al*., 2007; Liu *et al.,* 2010). These studies used transgenic *Arabidopsis* and investigated the relationship between gene expression and disease resistance in the above‐ground tissues.

Considering SA as a major component of SAR, we examined the expression of the SAR marker genes *PR1* and *PR3* in the transgenic soybean. No significant difference in mRNA abundance of *GmPR1* and *GmPR3* was detected between the transgenic and the nontransgenic control lines (Figure S3), which is consistent with our prior hairy root study (Lin *et al*., 2013). In other studies using transgenic plants, varied PR gene expression was found, such as the increased expression of *PR1* and *PR3* in transgenic soybean overexpressing *GmEREBP1*, a transcription factor activating defence genes (Mazarei *et al*., 2007), 10% down‐regulated *PR1* and 5‐fold increased *PR3* expression in transgenic hairy roots overexpressing *GmSNC1*, an SAR‐related gene (Maldonado *et al*., 2014). Our results may indicate that overexpression of *GmSAMT1* in soybean could contribute to the broad‐spectrum resistance observed for at least for the three SCN HG types tested here, but not through altering the steady‐state levels of PR gene expression.

Plant tissues infected by nematodes are highly metabolically active (Hofmann *et al*., 2010). Through analysis of several important metabolites in the SA biosynthesis pathway, intriguing alteration of some compounds in our transgenic soybean was observed, such as decreased SA and BA and increased PA in leaves (Figure 5). The most parsimonious explanation of our results is that *GmSAMT1* overexpression caused SA to decrease, subsequently impacting pools of its direct putative precursor, BA, presumably through a feedback regulatory loop. PA, as one important component in SA biosynthesis, was suggested to be regulated by chorismate mutase (Bekal *et al*., 2003; Huang *et al*., 2005; Jones *et al*., 2003; Vanholme *et al*., 2009). Plant‐parasitic cyst and root‐knot nematodes have been shown to synthesize and secrete chorismate mutases into plant cells and tissues, presumably to manipulate SA and basal defence responses (reviewed in Hewezi, 2015). Overexpression of *SAMT* may drive the biosynthesis of PA for increased resistant response to SCN infection. While we did perform metabolite analysis on the transgenic soybean roots, metabolite levels were below detection. Basal SA levels vary among plant species and tissues within the same plant species (Cho *et al*., 2013). No MeSA was detected in leaf and root tissues of transgenic and control plants. MeSA might be synthesized and emitted from plants in the volatile form. Overall, the change of metabolites in transgenic soybean leaf may lead to a nutritional deficiency of SCN, hinder the nematode development and increase the transgenic soybean resistance against SCN.

In addition to its role in defence, SA is involved in the regulation of plant growth and development (Gutiérrez‐Coronado *et al*., 1998). In *A. thaliana*, SA‐deficient *NahG* transgenic lines and *sid2* mutants displayed increased growth and seed production compared with wild‐type plants (Abreu and Munné‐Bosch, 2009). Therefore, we examined the possible effect of SCN stress on growth of transgenic soybean plants. At 35 days postinoculation of SCN, the transgenic soybean line L16‐2 had significantly higher shoot weight and height than the nontransgenic control soybean (Figure 4). This trend was also observed in other transgenic soybean lines, which suggests that the transgenic plants may adapt to SCN stress by increased vegetative growth. When growing under relatively warmer greenhouse conditions, which might have included more heat stress, transgenic soybean lines produced higher seed yield and number of seeds per plant than nontransgenic soybean (Figure S5). However, there was no significant difference of seed weight and number between transgenic soybean lines and nontransgenic soybean at 22 °C (Figure S4). This suggests that the transgenic plants may be better acclimated to the less desirable (i.e. hotter) environment. It has been reported that SA plays a role in photosynthetic heat stress tolerance by induced proline accumulation and interaction with ethylene (Khan *et al*., 2013). Congruent results were observed under field conditions (Table 1, Figure S6).

In summary, transgenic *GmSAMT1* soybean appears to have prospects as a useful crop‐based tool to manage SCN in the field. Moreover, as *GmSAMT1* is a soybean gene, it is a good candidate for using a cisgenic approach for deployment, which might decrease the regulatory burden for commercialization. Furthermore, tuning the expression of *GmSAMT1* might confer resistance to pathogens other than SCN. A broad‐based pathogen‐resistant cisgenic soybean plant would have value to a broad geographic range of soybean growers.

## Experimental procedures

### Transgenic soybean plants

The cassette of 35S promoter:GmSAMT1:NOS terminator from pJL‐OFP‐35S:GmSAMT1 plasmid (Lin *et al*., 2013) was excised by digestion with *EcoR*I and *Hind*III and was cloned into the corresponding sites of the binary vector pTF101.1 (Paz *et al*., 2006). The binary construct was introduced into soybean cv. ‘Williams 82’ using *Agrobacterium*‐mediated transformation by the Iowa State University Plant Transformation Facility (Ames, USA) (Paz *et al*., 2006).

### Plant cultivation

Soybean transformants (T0) were selected using Liberty^®^ herbicide with glufosinate‐ammonium as active ingredient (Bayer CropScience, Research Triangle Park, NC) (Paz *et al*., 2006) and allowed to self‐pollinate in the greenhouse. T1 progeny of these transformants were selected using Finale^®^ herbicide with glufosinate‐ammonium as active ingredient (Bayer CropScience, Research Triangle Park, NC) for selection and 3 : 1 (resistant:susceptible) segregating lines were selected and self‐pollinated in a greenhouse. Progeny (T2) of the individual T1 plants were observed for Finale^®^ herbicide selection and those that showed 100% resistance to Finale^®^ herbicide were selected (homozygous lines). T3 homozygous lines were used in all assays described here. To determine whether the observed segregation ratios deviated significantly from the expected segregation ratios, a chi‐square (χ^2^) test was undertaken with an alpha level of 0.05 using R software (version 3.1.0) (R Foundation for Statistical Computing, Vienna, Austria).

Soybean plants were grown in 4L pots containing 3B Mix (Fafard, Bradenton, FL) and fertilized with Osmocote (Scotts, Marysville, OH) in two greenhouses. The environmental conditions of the cool greenhouse were 16‐h day length and 22 °C day/night temperature, whereas the warmer greenhouse was 14‐h day length and 27 °C for day/23 °C for night temperatures. The mean value of seed number per plant, seed weight per plant and single seed weight was calculated from four biological replicates of each line. Statistical significance was tested with one‐way analysis of variance (ANOVA) followed by a Fisher's least significant difference (LSD) test with an alpha level of 0.05 using R software (version 3.1.0).

### Examination by PCR and copy number determination

The insertion of *GmSAMT1* and *Bar* (bialaphos resistance gene) genes was examined by PCR using genomic DNA as template. Genomic DNA was extracted from leaves of homozygous T3 transgenic soybeans by CTAB method (Stewart and Via, 1993). Genomic DNA was diluted to 20 ng/μL for PCR. The PCR conditions were as follows: 94 °C for 5 min followed by 35 cycles at 94 °C for 30 s, 56 °C for 30 s and 72 °C for 30 s and a final extension at 72 °C for 10 min. To only amplify the transgene *GmSAMT1*, a specific forward primer was designed to locate on the ending region of *GmSAMT1* and a reverse primer was located at the beginning region of NOS terminator as follows: Transgene‐SAMT‐F (forward): 5′‐CTACCAGCAAATCTTGGCTGAA‐3′ and Transgene‐SAMT‐R (reverse): 5′‐GCAAGACCGGCAACAGGA‐3′. The same pair of primers was also used for following genomic qPCR (for determining the copy number) and qRT‐PCR (for determining the expression level) of the transgene *GmSAMT1*. The primers for amplifying *Bar* gene are as follows: Bar‐F (forward): 5′‐CGAGTCGACCGTGTACGTC‐3′ and Bar‐R (reverse): 5′‐GCAACTGTCGGTCCAATAGAC‐3′. To determine the copy number of the transgene *GmSAMT1*, real‐time qPCR with internal reference gene‐based method was performed as described (Mason *et al*., 2002; Yuan *et al*., 2007). Four 1 : 5 serial dilutions were made from 20 ng/μL genomic DNA to generate standard curves. Ct values and relative abundance were calculated using software supplied with the Applied Biosystems 7900 HT Fast Real‐Time PCR system. DNA accumulation was measured using SYBR Green (Applied Biosystems, Foster City, CA) as the reference dye. The soybean ubiquitin 3 gene (GmUBI, GenBank accession D28123) was used as an internal reference gene. The sequences of gene‐specific primers were as follows: GmUBI‐F (forward): 5′‐GTGTAATGTTGGATGTGTTCCC‐3′ and GmUBI‐R (reverse): 5′‐ACACAATTGAGTTCAACACAAACCG‐3′. The following qPCR conditions were used: 50 °C for 2 min; 95 °C for 10 min; followed by 40 cycles of 95  C for 15 s; 60 °C for 1 min; 72 °C for 30 s. All the assays were conducted in duplicate. PCR efficiencies for transgene and reference gene were equal among the samples. The copy number for each transgenic soybean lines was calculated from three samples with 95% confidence intervals.

### Expression analysis by quantitative reverse transcription PCR (qRT‐PCR)

Quantitative reverse transcription PCR (qRT‐PCR) was performed as previously described (Lin *et al*., 2013) to assay *GmSAMT1* expression in roots. Relative transcript abundance of soybean PR genes (*GmPR1* and *GmPR3*), which are associated with SA signalling (Mazarei *et al*., 2007), was also examined in these transgenic soybean plants. Total RNA was isolated from soybean root tissue using TRIzol Reagent (Molecular Research Center, Cincinnati, OH) following the manufacturer's instructions. RNA was treated with RNase‐Free DNase set (Qiagen, Hilden, Germany) to remove genomic DNA and cleaned on the RNeasy columns (Qiagen) following the manufacturer's instructions. Total RNA (about 1 μg) was reverse‐transcribed to synthesize cDNA using High‐Capacity cDNA Reverse Transcription Kit (Applied Biosystems) for qRT‐PCR according to the manufacturer's instructions. First‐strand cDNA was diluted and placed in each qRT‐PCR reaction. DNA accumulation was measured using SYBR Green as the reference dye with GmUBI as a internal reference gene. The sequences of gene‐specific primers for endogenous *GmSAMT1*, transgene *GmSAMT1*, total *GmSAMT1*,* GmUBI*,* GmPR1* and *GmPR3* are listed in Table S3. Statistical significance was tested with a one‐way ANOVA followed by Fisher's LSD with an alpha level of 0.05 using R software (version 3.1.0).

### SCN inoculation

SCN eggs of HG Type 1.2.5.7 (race 2), HG Type 0 (race 3) and HG type 2.5.7 (race 5) were utilized for SCN assay. SCN eggs were purified by centrifugal flotation in a sucrose solution (35%) and cleaned in 10 mm MES buffer (pH 6.5). Then, the eggs were hatched on no. 500‐mesh (25 μm) sieve using 3.14 mm ZnSO_4_ as hatching solution at 25 °C. Preparasitic second‐stage juveniles (J2s) were collected and suspended in 0.1% agarose solution at a concentration of 2000 J2/mL, and 0.5 mL of J2 inoculum was applied to each soybean root of 3‐day‐old seedlings using a 1 mL pipette (1000 J2/plant). The inoculation was undertaken in a growth chamber for 2 days with 16‐h day length and 25 °C day/night temperature. The soybean seedlings were transplanted to sterile sand in 50 cm^3^ cone‐tainers (12 cm in length, 2.5 cm inside diameter) randomly arranged within the tray (Stuewe and Sons, Tangent, OR). The SCN‐inoculated soybeans were maintained for 35 days in the growth chamber with 16‐h day length and 22 °C day/night temperature and 100–110 μmol/cm/s light intensity. Plants were watered every 2 days and fertilized weekly with Peters Professional fertilizer (Scotts, Marysville, OH).

### Bioassay of SCN‐inoculated transgenic soybeans

At 35 days postinoculation, the root samples in the cone‐tainers were collected and kept at 4 °C for cyst counting. The SCN bioassay was conducted following the protocol described by Kandoth *et al*. (2013). Root samples and sand were gently moved to a beaker from the cone‐tainer after soaking in the water for a few minutes. The cysts were dislodged from the root samples using a water sprayer and cysts existed in the sand were collected on top of a no. 20‐mesh (750 μm) sieve stacked on top of a no. 60‐mesh (250 μm) sieve to collect SCN females/cysts. Cysts were rinsed into the 50‐mL tube and manually counted under a dissecting microscope. SCN eggs collected from each soybean line were released from cysts by gently grinding the cysts with a rubber stopper on a set of two sieves (top sieve no. 200 and bottom sieve no. 500) for counting. The female index was calculated for each soybean line where the average number of mature female nematodes and cysts on the transgenic soybean line was divided by the average number of mature female nematodes and cysts on the susceptible soybean control line (‘Williams 82′) and multiplied by 100. The result was combination of three independent experiments with at least four plants analysed in each bioassay. For SCN HG type 2.5.7 (race 5) inoculated soybean, the fresh shoot weight, shoot height and numbers of internodes were measured for individual plants (*n* ≥ 8). Statistical significance was tested with one‐way ANOVA followed by Fisher's LSD with an alpha level of 0.05 using R software (version 3.1.0). To study the association between the gene expression and female index, Pearson's product‐moment correlation coefficient was calculated using R software (version 3.1.0).

### Metabolite analysis: salicylic acid, benzoic acid and phenylalanine

A modified gas chromatography–mass spectrometry (GC‐MS) metabolite analysis was performed as previously described (Yang *et al*., 2009) to measure the key compounds of transgenic soybean leaves. Approximately 100‐mg leaf tissue that were ground in liquid nitrogen were quantitatively transferred to scintillation vials containing 80% ethanol (aqueous) and sorbitol (0.100 g/100 mL) as an internal standard to correct for sample loss during extraction and heating and differences in derivatization efficiency. After two successive 24‐h extraction with 80% ethanol, the supernatants were combined, and a 1 mL aliquot was dried down in a helium stream, then dissolved in silylation‐grade acetonitrile, followed by the addition of *N*‐methyl‐*N*‐trimethylsilyltrifluoroacetamide (MSTFA) with 1% trimethylchlorosilane (Pierce Chemical Co., Rockford, IL), and then heated for 1 h at 70 **°**C to generate trimethylsilyl derivatives. After 2 days, 1 mL aliquots were injected into a ThermoFisher DSQII GC‐MS, fitted with an Rtx‐5MS (cross‐linked 5% PH ME Siloxane) 30 m × 0.25 mm × 0.25 μm film thickness capillary column (Restek, Bellefonte, PA). The amounts of salicylic acid (SA), benzoic acid (BA) and phenylalanine (PA) were calculated by area integration, and relative concentrations were determined based on the quantity of the internal standard (sorbitol). The SA, BA and PA peaks were quantified by extracting m/z 267, 179, 218, respectively, to minimize integration of co‐eluting metabolites. The concentrations were estimated using a correction factor to scale from the extracted ion to the total ion because of skewed peak shapes or additional m/z fragments indicative of peak overlap. The correction factors were predetermined from the standards of SA, BA and PA. The mean concentration of the each metabolite was calculated from two biological replicates. Statistical significance was determined by Student's *t*‐test with an alpha level of 0.05 using R software (version 3.1.0). To study the association between the gene expression and SA, BA and PA level, Pearson's product‐moment correlation coefficient was calculated using R software (version 3.1.0).

### Field evaluation of transgenic soybeans

A 1‐year field experiment was performed under USDA APHIS BRS permit to test plant performance at the East Tennessee Research and Education Center, ETREC, Plant Science Farm (Knoxville, Tenn.) in which there was negligible SCN pressure (there were 0–3 cysts per 100 mL soil before planting). The genotypes tested were comprised of five transgenic lines and two control lines. One control was the nontransgenic ‘Williams 82’, and the other control was a null segregant line. The field experiment was set in a randomized complete block design with three replications. The experimental unit consisted of two 3.7 m rows spaced 76 cm apart each containing 84 plants. The experiment was evaluated throughout the season for phenotypic traits. Flower colour was recorded as described by Fehr *et al*. (1971) when 95% of the plants were in bloom (R1–R2 stages). Pubescence colour and maturity were recorded at R8 stage when 95% of the pods were mature. Plant height was measured as the average plant height from the base of the stem at the soil surface to the top of the plant at maturity. Lodging was scored at maturity with a score range from one (all plants erect) to five (all plants prostrate). The plants were cut at harvest and threshed with a small‐plot thresher (Almaco, Nevada, IA). Three weeks after harvest moisture ratings were taken using a bench grain moisture tester (Dickey‐John, Auburn, IL), and seed weight was measured. For data analysis, seed yield was calculated on a 100% dry weight basis.

## Supporting information


**Figure S1** Vector and PCR results for *GmSAMT1* transgenic soybean.
**Figure S2** The standard curves used for determining the transgene (*GmSAMT1*) copy number with internal reference gene (*GmUBI*).
**Figure S3** Relative transcript levels of (a) *GmPR1* and (b) *GmPR3* by qRT‐PCR normalized to the expression of *GmUBI*.
**Figure S4** Seed weight and seed number of T3 homozygous transgenic soybean plants overexpressing *GmSAMT1* grown in the greenhouse with 22 °C day/night temperature.
**Figure S5** Seed weight and seed number of T3 homozygous transgenic soybean plants overexpressing *GmSAMT1* grown in the greenhouse with 27 °C for day/23 °C for night temperatures.
**Figure S6** Transgenic soybean grown in the field in 2015 in Knoxville, Tenn., USA.
**Table S1** Finale^®^ herbicide resistance segregation analysis of T1 progeny of 10 transgenic soybean lines.
**Table S2** Confirmation of the copy number of the T2 homozygous transgenic soybean lines via genomic qPCR.
**Table S3** List of primers used in qRT‐PCR.
