# Supplementary material for: Transgenic soybean overexpressing GmSAMT1 exhibits resistance to multiple‐HG types of soybean cyst nematode Heterodera glycines
Source: Plant Biotechnol J. 2016 May 23;14(11):2100–9. doi: 10.1111/pbi.12566 (PMC5095773; doi:10.1111/pbi.12566)

**SUPPORTING INFORMATION**

**
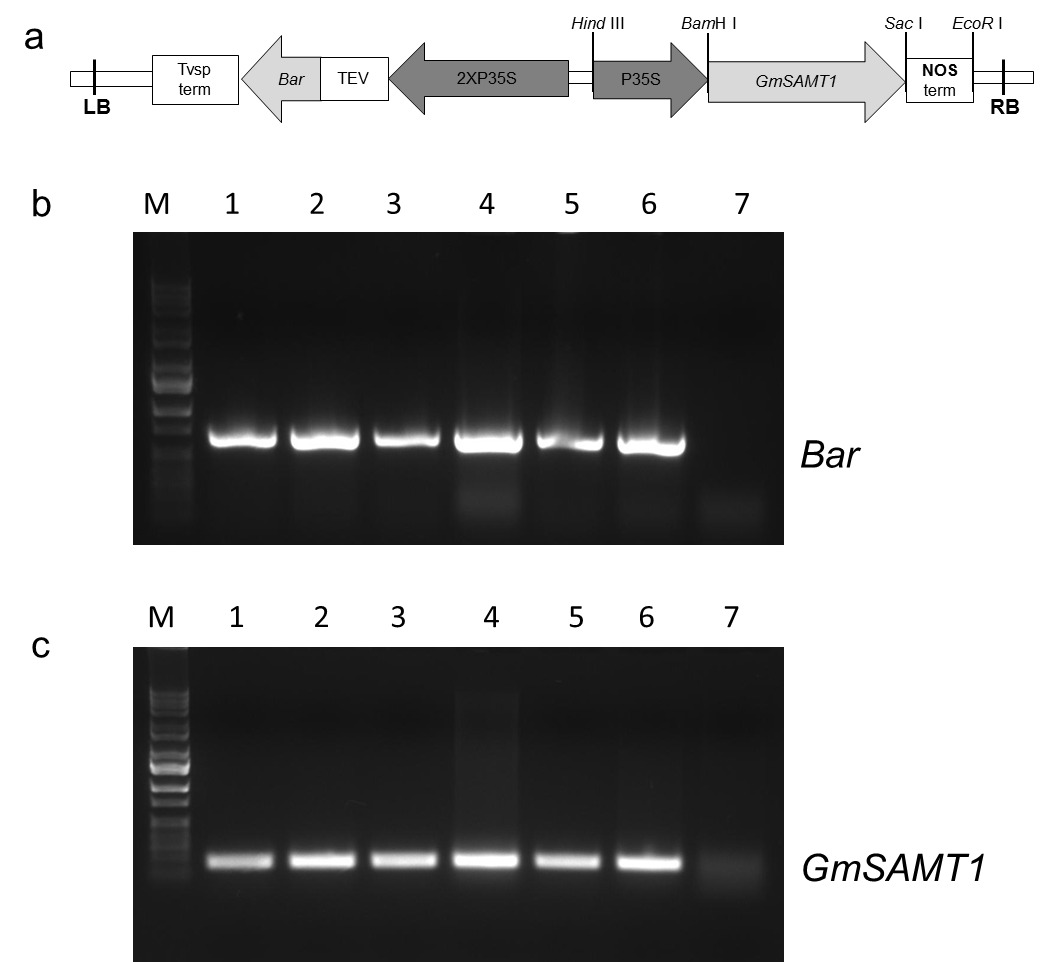
**

**Figure S1** Molecular characterization of transgenic soybean plants overexpressing *GmSAMT1* gene. (a) pTF-GmSAMT1 vector construct used for overexpression of *GmSAMT1*. (b) Genomic PCR confirming the insertion of *Bar* gene (bialaphos resistance gene). (c) Genomic PCR confirming the insertion of the *GmSAMT1* gene. M: DNA marker. Lane 1 to 6 are for transgenic soybean lines L5-5, L16-2, L16-3, L20-3, L56-3, and L65-3. Lane 7 is for non-transgenic Williams 82 soybean control, no amplification was observed in DNA samples from the control plants.


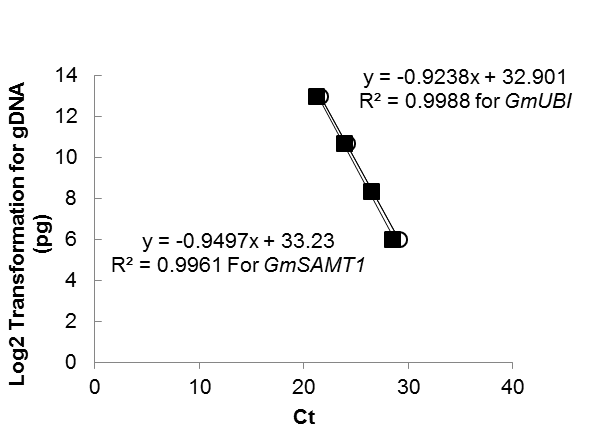


**Figure S2** The standard curves used for determining the transgene (*GmSAMT1*) copy number with internal reference gene (*GmUBI*).

**
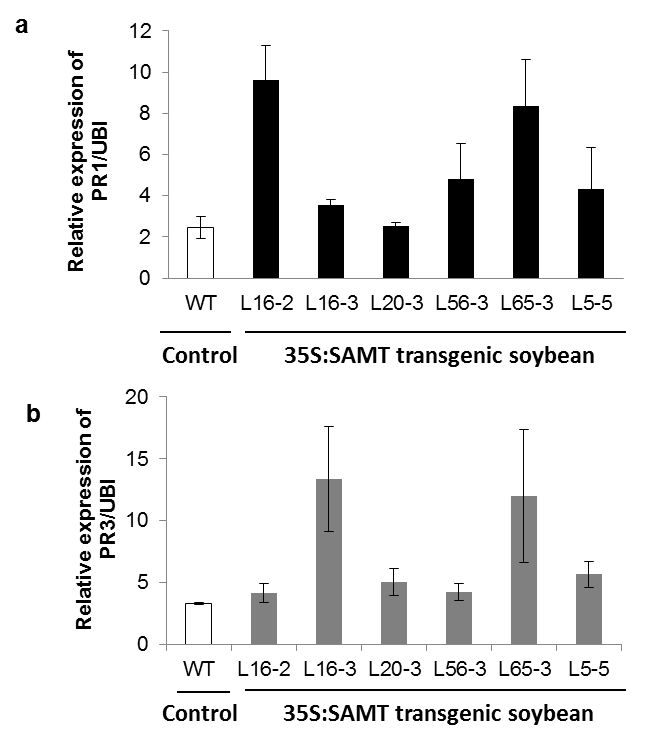
**

**Figure S3** PR gene expression level by qRT-PCR. (a) The relative expression of *GmPR1*. (b) The relative expression of *GmPR3*. The examined genes were normalized to the expression of *GmUBI*. Bars represent mean values of three biological replicates ± standard error. There was no significant difference on PR gene expression between the transgenic lines and the control line. Non-transgenic Williams 82 (WT) soybean as control and 35S:SAMT T3 homozygous transgenic soybean lines L5-5, L16-2, L16-3, L20-3, L56-3, and L65-3 with overexpression of *GmSAMT1* were studied.

**
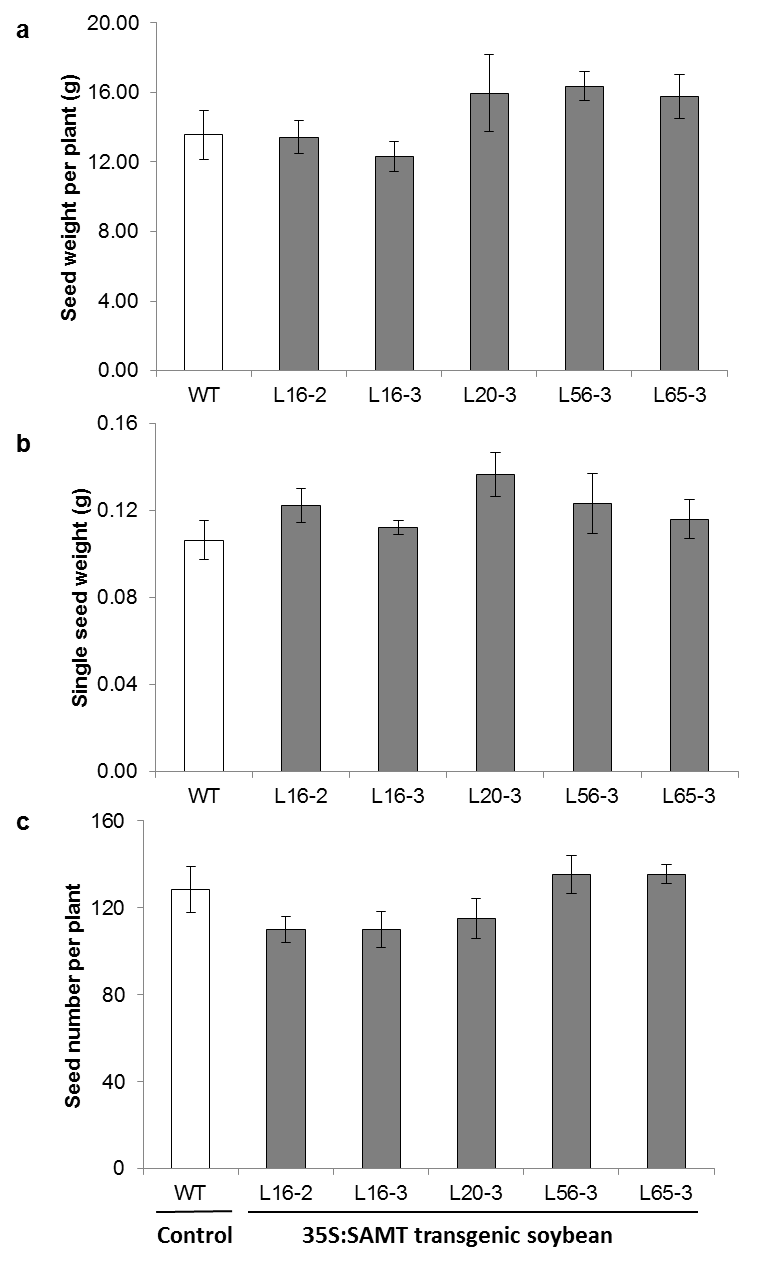
**

**Figure S4** Seed weight and seed number of T3 homozygous transgenic soybean plants overexpressing *GmSAMT1* grown in the greenhouse with 22°C day/night temperature. Seed weight per plant (a), single seed weight (b), and seed number per plant (c). Bars represent mean values of four biological replicates with ± standard error. Non-transgenic Williams 82 (WT) soybean as control and 35S:SAMT transgenic soybean lines L16-2, L16-3, L20-3, L56-3, and L65-3 with overexpression of *GmSAMT1* were studied.

**
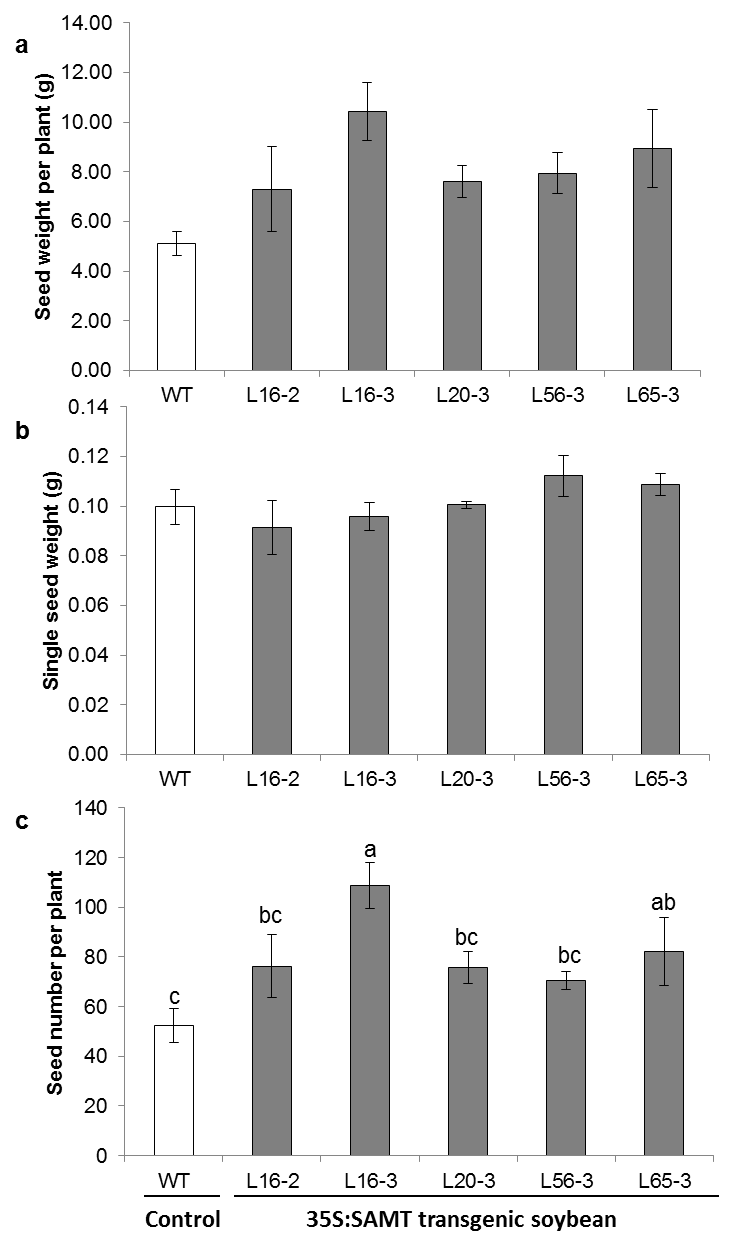
**

**Figure S5** Seed weight and seed number of T3 homozygous transgenic soybean plants overexpressing *GmSAMT1* grown in the greenhouse with 27°C for day/ 23°C for night temperatures. Seed weight per plant (a), single seed weight (b), and seed number per plant (c). Bars represent mean values of four biological replicates with ± standard error. Bars with different letters are significantly different at *P* < 0.05 as tested by Fisher’s least significant difference. Non-transgenic Williams 82 (WT) soybean as control and 35S:SAMT transgenic soybean lines L16-2, L16-3, L20-3, L56-3, and L65-3 with overexpression of *GmSAMT1* were studied.

**
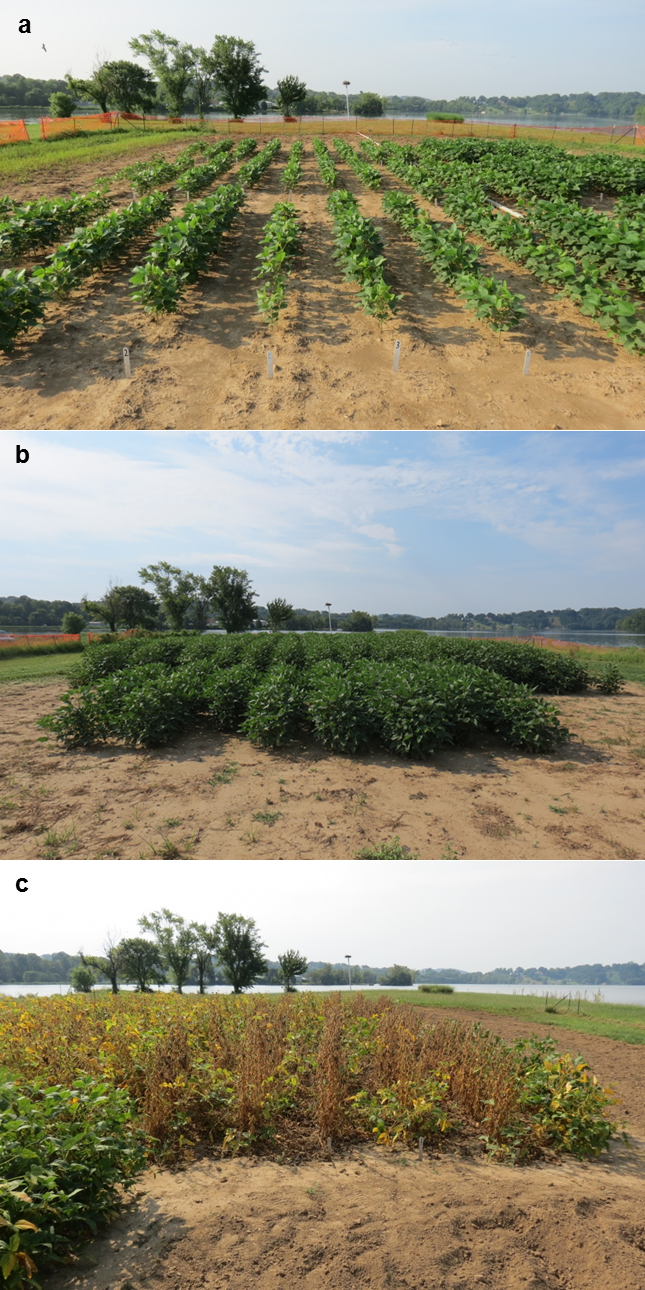
**

**Figure S6** Transgenic soybean grown in the field. (a) One-month old soybean (b) Two-month old soybean (c) Four-month old soybean. No obvious phenotype difference was observed between the transgenic soybean and non-transgenic soybean.

**Table S1** Finale® resistance segregation analysis of T1 progeny of 10 transgenic soybean lines.

| **T1 line** | **# resistant plant** | **# sensitive plant** | **Predicted ratio** | **p-value** | **Predicted copy number** |
| --- | --- | --- | --- | --- | --- |
| L3 | 14 | 5 | 3:1 | 0.895 | 1 |
| L5 | 20 | 5 | 3:1 | 0.564 | 1 |
| L16 | 15 | 6 | 3:1 | 0.705 | 1 |
| L20 | 18 | 10 | 3:1 | 0.190 | 1 |
| L31 | 26 | 1 | 15:1 | 0.585 | 2 |
| L35 | 16 | 11 | 3:1 | 0.059 | 1 |
| L39 | 10 | 7 | 3:1 | 0.123 | 1 |
| L56 | 20 | 4 | 3:1 | 0.346 | 1 |
| L59 | 16 | 5 | 3:1 | 0.900 | 1 |
| L65 | 13 | 7 | 3:1 | 0.302 | 1 |

A chi-square (χ^2^) analysis was undertaken to determine whether the observed segregation ratios deviated significantly from the expected segregation ratios (*P*<0.05).

**Table S2** Confirmation of the copy number of the T2 homozygous transgenic soybean lines via qPCR.

| **T2 Line** | **Copy number** | **Confidence interval for mean of copy number** | **Predicted copy number** |
| --- | --- | --- | --- |
| L16-2 | 1.83 | (1.25, 2.40) | 2 |
| L16-3 | 1.52 | (0.20, 2.84) | 2 |
| L20-3 | 0.94 | (0.56, 1.33) | 1 |
| L56-3 | 0.87 | (0.72, 1.01) | 1 |
| L65-3 | 0.82 | (0.54, 1.10) | 1 |
| L5-5 | 1.04 | (0.70, 1.38) | 1 |

**Table S3** List of primers used in qRT-PCR.


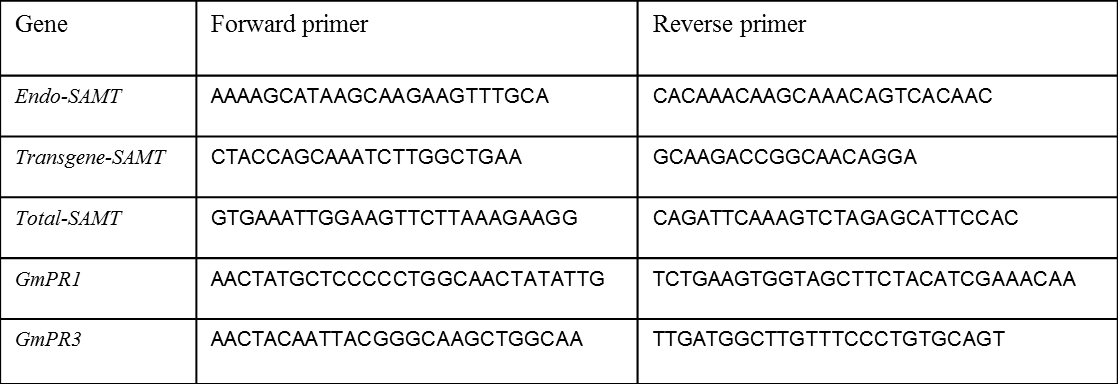

Supplement: Supplementary file 1 — Figure S1 Vector and PCR results for GmSAMT1 transgenic soybean. Figure S2 The standard curves used for determining the transgene (GmSAMT1) copy number with internal reference gene (GmUBI). Figure S3 Relative transcript levels of (a) GmPR1 and (b) GmPR3 by qRT‐PCR normalized to the expression of GmUBI. Figure S4 Seed weight and seed number of T3 homozygous transgenic soybean plants overexpressing GmSAMT1 grown in the greenhouse with 22 °C day/night temperature. Figure S5 Seed weight and seed number of T3 homozygous transgenic soybean plants overexpressing GmSAMT1 grown in the greenhouse with 27 °C for day/23 °C for night temperatures. Figure S6 Transgenic soybean grown in the field in 2015 in Knoxville, Tenn., USA. Table S1 Finale® herbicide resistance segregation analysis of T1 progeny of 10 transgenic soybean lines. Table S2 Confirmation of the copy number of the T2 homozygous transgenic soybean lines via genomic qPCR. Table S3 List of primers used in qRT‐PCR. [file PBI-14-2100-s001.docx]
